# Body-Related Attentional Bias in Adolescents Affected by Idiopathic Scoliosis

**DOI:** 10.3390/ejihpe13090138

**Published:** 2023-09-18

**Authors:** Margherita Bertuccelli, Maria Rubega, Francesca Cantele, Claudia Favero, Andrea Ermolao, Emanuela Formaggio, Stefano Masiero

**Affiliations:** 1 Padova Neuroscience Center, University of Padova, 35131 Padova, Italy; stef.masiero@unipd.it; 2 Department of Neuroscience, Section of Neurology, University of Padova, 35128 Padova, Italy; 3 Department of Neuroscience, Section of Rehabilitation, University of Padova, 35128 Padova, Italy; maria.rubega@unipd.it (M.R.); francesca.cantele.11@gmail.com (F.C.); emanuela.formaggio@unipd.it (E.F.); 4 Clinical Network of Sports and Exercise Medicine of the Veneto Region, 35131 Padova, Italy; claudia.favero.2@gmail.com (C.F.); andrea.ermolao@unipd.it (A.E.); 5 Sport and Exercise Medicine Division, Department of Medicine, University of Padova, 35128 Padova, Italy

**Keywords:** body image, adolescence, information processing biases, scoliosis

## Abstract

Attentional biases toward body-related information increase body dissatisfaction. This can lead at-risk populations to develop psychopathologies. This phenomenon has not been extensively studied in girls affected by idiopathic scoliosis. This work aimed to study the cognitive processes that could contribute to the worsening and maintaining of body image disorders in adolescent idiopathic scoliosis. Twenty-eight girls were recruited and tested for body image dissatisfaction through the Scoliosis-Research-Society-22-revised (SRS-22r) questionnaire. Attentional biases towards disease-related body parts were assessed using a computerized visual match-to-sample task: girls were asked to answer as fast and accurately as possible to find the picture matching a target by pressing a button on a computer keyboard. Reaction times (RTs) and accuracy were collected as outcome variables and compared within and between groups and conditions. Lower scores in SRS-22r self-image, function, and total score were observed in scoliosis compared to the control group (*p*-value < 0.01). Faster response times (*p*-value = 0.02) and higher accuracy (*p*-value = 0.02) were detected in the scoliosis group when processing shoulders and backs (i.e., disease-relevant body parts). A self-body advantage effect emerged in the scoliosis group, showing higher accuracy when answering self-body stimuli compared to others’ bodies stimuli (*p*-value = 0.04). These results provide evidence of body image dissatisfaction and attentional bias towards disease-relevant body parts in girls with scoliosis, requiring clinical attention as highly predisposing to psychopathologies.

## 1. Introduction

The appearance of body image and the subjective perception of our own body are crucial factors in determining self-confidence. This is particularly true for adolescent girls. Body image is a multidimensional concept including subjective perception, emotional, and cognitive-related body aspects [1]. Each of these components can be affected [2], resulting in different psychopathologies: subjective perceptual alterations of the body are observed in body dysmorphic disorders [3], while negative body-related emotions can lead to depression [4], or unhealthy behaviors such as substance abuse [5] and eating disorders [6]. Cognitive processes can play a role in triggering and maintaining the above-mentioned disorders. Selective attention to body shape and weight-related information can increase body image dissatisfaction in eating disorders [7]. Additionally, it was observed that even among healthy subjects, it is possible to induce vulnerability to body dissatisfaction if trained to attend to body-related information [8]. Attentional biases are described as the tendency to prioritize the processing of specific stimuli compared to others [7,9]. They can result from faster engagement or difficult disengagement with the relevant stimulus [9]. However, in some cases, even a systematic faster disengagement from a stimulus can be a sign of attentional bias (i.e., attentional avoidance [10]). Attentional biases and their association with body dissatisfaction have been extensively studied in eating disorders, body dysmorphic disorders, and substance abuse conditions [11,12,13,14]. Nonetheless, several other clinical populations can be affected by similar types of attentional disorders associated with body image dissatisfaction. Among them, adolescents affected by idiopathic scoliosis have been neglected.

Adolescent idiopathic scoliosis (AIS) is a widely diffused clinical condition that affects 1–3% of the adolescent population [15]. Clinically, AIS is characterized by spinal deviation in the frontal plane (>10 Cobb degrees) and the concurrent rotation of the affected vertebral bodies [15]. Considering both the disfiguring appearance caused by spinal deformity and the adolescence period being a predisposing factor per se to body image dissatisfaction [16], girls with AIS are particularly at risk of developing body-image-related disorders [17]. Indeed, many studies report high rates of body image dissatisfaction in this clinical population, mostly assessed by administering self-reported questionnaires [18] and rated as the major psychological effect of AIS [19]. In this regard, a recent literature review emphasized the spectrum of psychological disturbances often associated with AIS, which include body image alterations, eating disorders, and mood disorders [20]. Particularly, it has been reported that up to 50% of girls with AIS report social limitations and low self-esteem due to their physical appearance compared to 15% of healthy adolescents [20,21,22]. This can be due to traditional treatments of scoliosis implying the adoption of noticeable braces, which have a great impact on girls’ self-esteem and quality of life [23]. Notably, body image disorders reported in AIS are not short-term phenomena but can persist even in adulthood [24].

AIS girls present on average, lower body weight, body mass index (BMI), and body fat percentage [25,26] tendencies, which seem to correlate with scoliosis severity [27]. Additionally, girls with scoliosis consistently demonstrate altered mood, anxiety, and depression [20].

Cognitive models of information processing have helped in disclosing the mechanisms underlying body image issues and psychopathologies in other clinical conditions [7]. However, to the authors’ knowledge, no previous study inquired about this aspect to better understand AIS psychopathological comorbidities. The novelty of this study lies in the assessment of possible information processing biases in AIS, which have been neglected by previous research.

Both clinicians and girls with scoliosis could benefit from the study of the cognitive dysfunctional processes which could contribute to the worsening and maintaining of body image disorders in AIS: the first, to have a deeper understanding of the psychopathological mechanisms associated with scoliosis and new therapeutic targets which could increase girls overall compliance with treatments and avoid developing any severe psychopathology; the latter may benefit from improved self-confidence and quality of life.

Due to the high prevalence of self-reported body image disorders and the spectrum of psychopathological conditions often reported in AIS, we hypothesize attentional biases towards disease-relevant body parts (i.e., shoulders, back) as dysfunctional mediating mechanisms predisposing to and/or maintaining these conditions, as happens in eating disorders and body dysmorphic disorders. Particularly, we expect (1) higher body image dissatisfaction compared with the healthy control group; (2) faster reaction times (RTs) and higher accuracy when answering to disease-relevant body parts compared with irrelevant ones (i.e., attentional bias); and (3) correlation between the level of body image dissatisfaction, RTs, and accuracy in answering to disease-relevant body parts.

## 2. Materials and Methods

This was a cross-sectional study conducted according to the criteria established by the Declaration of Helsinki. Parents’ written informed consent was collected before participating in the study. The project was approved by the Ethics Committee of the Teaching Hospital of Padova on 13 April 2023 (code: AOP2836).

### 2.1. Participants

Fourteen adolescent girls with a confirmed diagnosis of idiopathic scoliosis [15] were recruited from the Adolescence Spine Diseases Diagnostic and Therapeutic Centre of the Padova University Hospital. Girls were included according to their age (age range: 12–16 years), scoliosis Cobb angle (>20 degrees), and current treatment (i.e., brace and physiotherapy treatment). The control group included 14 adolescent girls (age range: 12–16 years) without diagnosed spinal pathology, enrolled at the Physical Activities Unit “Ai Colli” in the Social Health Department of the Padova Hospital. Exclusion criteria for both groups were a body mass index (BMI) greater than 25 or lower than 17 and playing sports at a competitive level, as both these factors can alter body image [28,29,30], and any known comorbid musculoskeletal, neurological, eating disorder and/or cognitive disorder. One girl of the control group and one girl with scoliosis were excluded for a BMI outside the inclusion range (see Table 1). Anamnestic information and clinical data for both groups were collected by an expert team of physicians (see Table 2).

### 2.2. Scoliosis Research Society-22 Revised

The Scoliosis Research Society-22 (SRS-22r) questionnaire is the gold standard self-reported measure of body image dissatisfaction in this clinical population. SRS-22r, Italian adaptation [31], comprises 22 questions scored 1 to 5, sorted into 5 domains (i.e., pain, self- image/appearance, mental health, function, and satisfaction with treatment). A total score ranging from 22 to 110 is also calculated, with lower scores indicating worse outcomes. In this paper, the SRS-22r results are expressed as the total sum of the scores divided by the number of items for each subdomain and total score (i.e., score range for each domain: 1–5).

### 2.3. Visual Match-to-Sample Task

**Figure 1:**
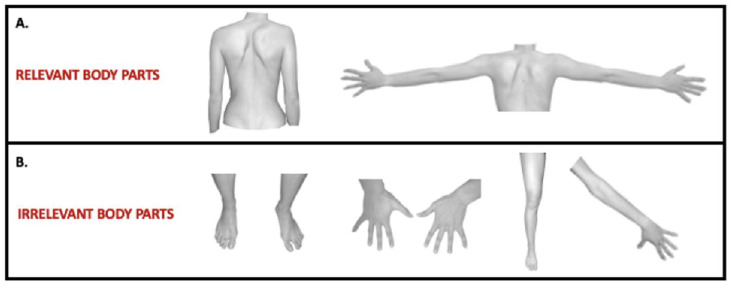
Disease-relevant and -irrelevant stimuli. Examples of disease-relevant ((**A**) panel) and -irrelevant pictures ((**B**) panel) used in the visual match-to-sample task.

A visual match-to-sample task (VMT) was developed by a re-adaptation of the paradigm used by Frassinetti [32]. The presentation and randomization of the stimulus were controlled using PsychoPy V3.0 software [33,34]. A total of 96 trials divided into three blocks were randomly presented to each participant. At the beginning of each trial, 3 pictures appeared vertically aligned and centered on a screen. Stimuli were pictures of body parts taken with a digital camera and modified by means of Adobe Photoshop software (Photoshop CC 2019 version 20.0.8) to appear as gray-scale images on a white background. Pictures displayed either disease-relevant (i.e., shoulders and backs) or -irrelevant (i.e., hands, feet, legs, arms) body parts (Figure 1).

Of the 96 trials, 24 displayed participant’s own body parts, while 72 held other people’s body parts. The participants were instructed to answer as accurately and as fast as possible the question “which picture matches the target?” by pressing with the index finger of their dominant hand the up and down arrows of a computer keyboard. No time limit was set to answer. To avoid automatic matching, the target pictures were presented upright in a red frame, while the up and down stimuli were randomly tilted 30° to the right or left. The 96 trials were organized in 3 blocks and preceded by a training block. Response accuracy (percentage of correct responses over the total number of trials), and RTs were collected as outcome variables. The task lasted overall 10 to 15 min.

### 2.4. Statistics

The data distribution was tested with a Shapiro–Wilks normality test. A two-sided Wilcoxon rank sum test or a two-sample *t*-test was performed according to the data distribution. Overall mean SRS-22r scores and subscores (i.e., pain, self-image/appearance, mental health, function, and satisfaction with treatment) were compared between groups. For the analysis of accuracy and RTs, the trials were clustered according to the target stimulus. In more detail, to test the effect of body part relevance, the trials were divided into:

- Relevant trials (trials presenting disease-relevant body parts as target) and irrelevant trials (disease-irrelevant body parts);
- Relevant-own trials (trials presenting disease-relevant body parts of the subject executing the task as target) and irrelevant-own trials (disease-irrelevant body parts of the subject executing the task).
- To test self-body advantage effect, the trials were divided into:
- Own body parts trials (trials presenting body parts of the subject performing the task, regardless of the relevance);
- Others’ body parts trials (trials presenting body parts of people other than the subject performing the task).

Within and between group comparisons of mean RTs and accuracy were performed among the conditions. Correlations between the SRS-22r questionnaire’s subscales and RTs and accuracy were assessed using the Pearson correlation coefficient (r). All statistical analyses were performed using RStudio software (Version 1.2.5001, RStudio Team, 2015).

## 3. Results

The controls and AIS groups were not different in terms of age (AIS µ ± σ = 14.87 ± 1.10; controls µ ± σ = 14.36 ± 1.18, t = 1.19, *p*-value = 0.24) and BMI (AIS median (IQR) = 20.01 (17.41–20.30); controls median (IQR) = 20.35 (19.07–21.31), W = 70, *p*-value = 0.21).

### 3.1. Scoliosis Research Society-22 Revised

**Figure 2:**
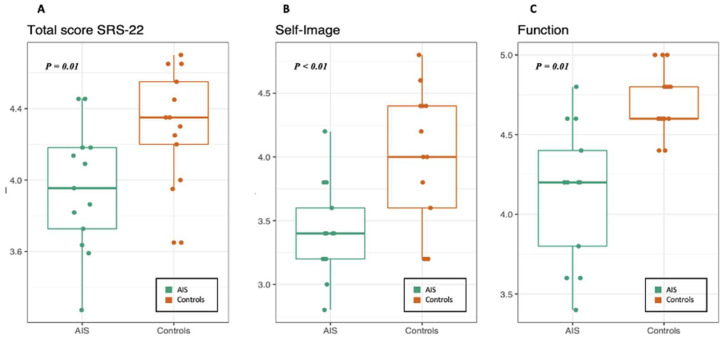
SRS-22r significant results. The boxplots represent SRS-22r average scores by group that were significantly statistically different. Lower scores were observed in the AIS group (green dots) compared to controls (orange dots) in the total score (**A**), and subdomains of self-image (**B**), and function (**C**).

The overall SRS-22r score for the AIS group was significantly lower than for the controls (AIS median (IQR) = 3.95 (3.72–4.18) vs. controls median (IQR) = 4.35 (4.20–4.55), W = 35, *p*-value = 0.01); see Figure 2. The subdomains analysis revealed significantly lower scores in the AIS group for the self-image domain (AIS median (IQR) = 3.40 (3.20–3.60)), compared to controls (median (IQR) = 4 (3.60–4.40), W = 36.5, *p*-value = 0.01) and the function domain (AIS median (IQR) = 4.20 (3.80–4.40) vs. controls median (IQR) = 4.60 (4.60–4.80), W = 18.5, *p*-value < 0.01); see Figure 2. The mental health and pain domains did not differ between groups (respectively, W = 62.5, *p*-value = 0.10, W = 94, *p*-value = 0.87). The mean scores of the domain “satisfaction with treatment” were not compared between groups as this domain was not applicable to the controls.

### 3.2. Visual Match-to-Sample Task

**Figure 3:**
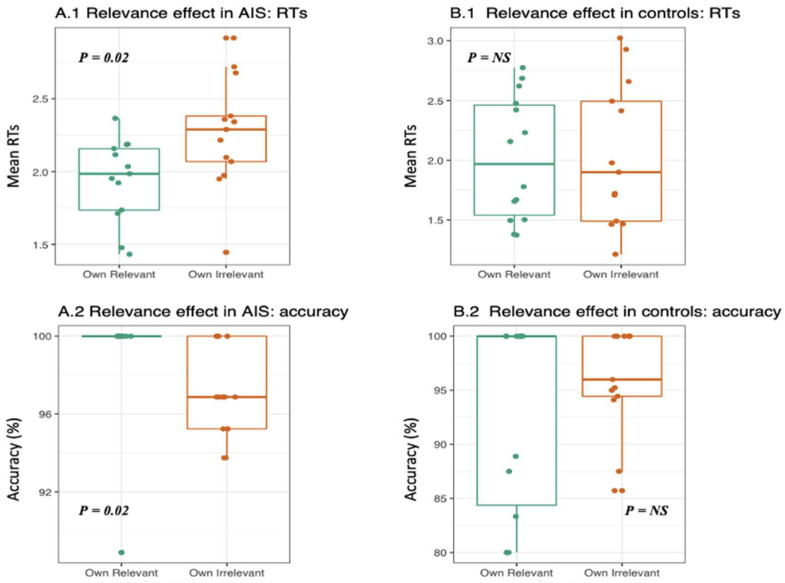
Body part relevance in AIS and controls. The boxplots represent average RTs and accuracy of AIS and controls in answering to their own relevant body parts (i.e., shoulders and backs—green dots) vs. their own irrelevant ones (i.e., arms, feat, legs, hands—orange dots). AIS group answered significantly (**A.1**) faster and (**A.2**) more accurately to their own relevant body parts compared to irrelevant ones. (**B.1**,**B.2**). No significant differences were observed in the control group.

AIS RTs to their own relevant body parts were faster compared to their own irrelevant ones (W = 41, *p*-value = 0.02); this effect was not observed in the control group (W = 90, *p*-value = 0.98; see Figure 3). In addition, AIS accuracy in answering to their own relevant body parts was higher compared to their own irrelevant ones (W = 99, *p*-value = 0.02), and this was not observed in the control group (W = 83.5, *p*-value = 0.71; see Figure 3). When comparing others’ relevant body parts with others’ irrelevant ones, no significant differences emerged for either the AIS or control groups. No significant differences were observed for both RTs and accuracy between the groups. However, a tendency towards higher accuracy was observed in the AIS group compared to controls when comparing the answers to their own relevant body parts (W = 95.5, *p*-value = 0.06).

**Figure 4:**
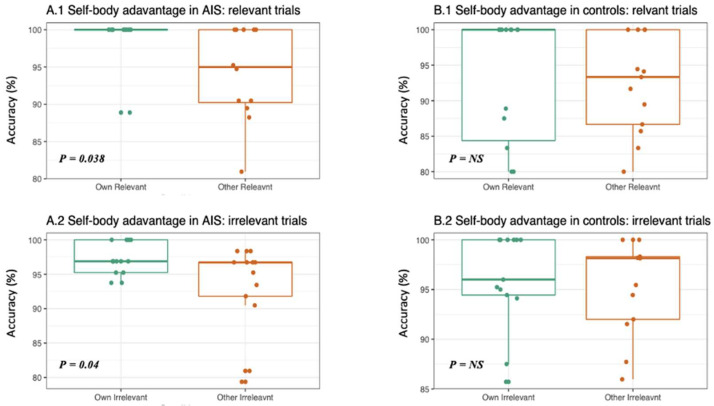
Self-body advantage effect in AIS and controls. Upper panels: The boxplots represent the accuracy of AIS (**A.1**) and controls (**B.1**) in answering to their own relevant body parts (i.e., shoulders and backs—green dots) vs. others’ relevant body parts (orange dots). (**A.1**) AIS group answered more accurately to their own relevant body parts compared to others’ ones. (**B.1**) No significant differences were observed in the control group. Lower panels: The boxplots represent (**A.2**) the accuracy of AIS and (**B.2**) controls in answering to their own irrelevant body parts (i.e., arms, feat, legs, hands—green dots) vs. others’ irrelevant body parts (orange dots). (**A.2**) AIS group answered more accurately to their own irrelevant body parts (green dots) compared to (**B.2**) controls.

The AIS group’s answer accuracy to their own relevant body part stimuli was higher compared to others’ relevant ones (W = 87.5, *p*-value = 0.038; see Figure 4) but no significant differences were observed in RTs (W = 90, *p*-value = 0.80). When comparing the answers to their own irrelevant and others’ irrelevant body part stimuli, the AIS group still have a self-advantage effect, showing higher accuracy to their own irrelevant images compared to others’ irrelevant ones (W = 123, *p*-value = 0.04; see Figure 4), but no differences in RTs emerged. No significant differences emerged in the control group for either accuracy or RTs (see Figure 4).

### 3.3. Correlations

The SRS-22r mental health score was positively correlated with the self-image score (r (11) = 0.65, *p* = 0.01). When considering their own relevant body part stimuli, no significant correlations emerged between accuracy, RTs, and questionnaire answers.

**Figure 5:**
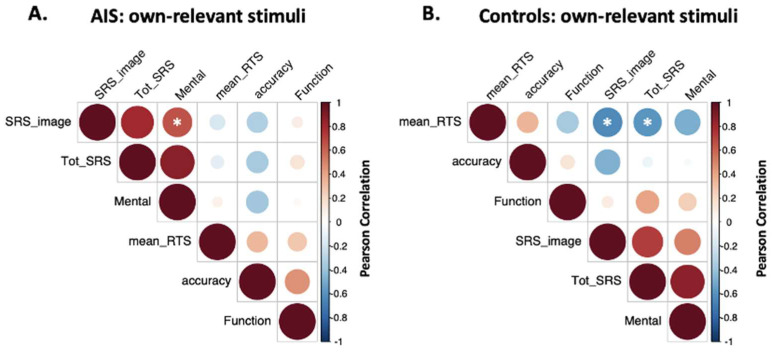
Correlation matrices**.** Correlation matrices between questionnaire scores (i.e., SRS_image: SRS-22r self-image domain; Tot_SRS: total score on SRS-22r; Mental: SRS-22r mental domain; Function: SRS-22r function domain), accuracy, and average RTs (i.e., mean_RTS). Subfigure **A** reports correlations for the AIS group, while subfigure **B** for the control group. Circles’ dimensions and colors identify the value of the Pearson correlation coefficient, i.e., largest dark red circle indicates maximum positive correlation (1), and largest dark blue circle represents maximum negative correlation (−1) between variables in the two axes. Asterisks indicate significant meaningful correlations.

In the control group, significant negative correlations were observed between mean RTs to their own relevant body part stimuli and self-image score (r (11) = −0.64, *p* = 0.01), and total score (r (11) = −0.59, *p* = 0.03), but no significant correlations between questionnaire scores and answers’ accuracy emerged (see Figure 5).

## 4. Discussion

This work provides evidence of attentional biases towards disease-relevant body parts and body image dissatisfaction in girls affected by idiopathic scoliosis. Body image dissatisfaction in our group was confirmed. Significantly lower scores in SRS-22r self-image, function, and total score were observed compared to control girls. This is in line with previous studies reporting body image dissatisfaction in this clinical population [20]. Additionally, SRS-22r self-image subscores were positively correlated with mental health subscores in the AIS group, suggesting their reciprocal dependence. Indeed, previous studies have linked negative self-image with a number of psychological and psychiatric symptoms in adolescence [35]. Particularly, body image concerns can be risk factors leading to mental health conditions such as anxiety, depression, eating disorders, unhealthy behaviors, and poorer quality of life [20]. These psychopathologies are highly comorbid with AIS: eating behavior disorders are the most reported ones, showing AIS girls having average lower BMI scores compared to controls [8]. Although not reaching statistical significance, this trend was also observed in our sample (AIS median (IQR) = 20.01 (17.41–20.30) vs. controls median (IQR) = 20.35 (19.07–21.31)). This lack of statistical significance could be due to the limited sample size of our study, as well as to the heterogeneity of scoliosis severity and the employed treatments.

A faster response time and higher accuracy were detected in the group of girls with AIS when they processed relevant body parts (i.e., shoulders and backs) compared to irrelevant ones, but only when the processed images were their own. This suggests specific attentional biases towards self-related shoulders and backs. These stimuli can probably engage attention more than other body parts in the AIS group as a direct consequence of their body image dissatisfaction. Indeed, previous studies proved the relation between a high-level of body image dissatisfaction and the tendency to allocate more attention to the own self-identified unattractive body parts [36].

Interestingly, we found a significant negative correlation between RTs with their own relevant own stimuli and the SRS self-image subscores of the SRS-22r questionnaire in the control group. This suggests that the control group’s girls with lower self-image scores tended to be slower in answering to their shoulders and backs. Possibly this could be a sign of attention disengagement/inefficient processing towards these specific body parts, as not related to their body dissatisfaction. A general effect of self-body advantage was also observed in the AIS group showing higher accuracy in answering to self-related pictures compared to others’ pictures, regardless of their relevance. The effect of self-body advantage is well recognized in the literature: it implies faster and more accurate recognition of self-body parts, as opposed to other people’s body parts, when implicitly processed [32]. It is noteworthy that pictures in our experiment were modified in a way that it was not possible for the participants to explicitly recognize their own body parts. A recent study inquiring as to the relation between self-body advantage and body image in younger and older women, shows that younger women have a greater self-body advantage compared with older ones, which is correlated with body image concerns [37]. Thus, the absence of self-body advantage effects in the control group may have been masked by both the small sample size and by a lack of body image concerns, as highlighted by their scores in the body image subitems of the SRS-22r. On the contrary, in the AIS group, the self-advantage effect may have been boosted by the attentional biases linked with their body image concerns.

Given the preliminary findings of this study pointing to a possible attentional bias of the AIS girls toward disease-related body parts, a larger cohort study would be useful to confirm this effect. Particularly, the study of attentional biases in AIS girls prior to any treatment would exclude the role of physical therapy and/or a brace in determining this effect. If confirmed, these results would suggest the importance of assessing AIS girls for information processing biases and the possibility to integrate scoliosis treatments with cognitive behavioral therapy (CBT) programs. Computer tests such as the one presented in this study are fast and easy to administer in clinical settings, as well as highly effective in catching attentional biases.

Acting on attentional biases in AIS girls would improve their general mental wellbeing by limiting body image concerns and, consequently, would increase their adherence to traditional treatments (i.e., braces and physiotherapy).

## 5. Limitations

The sample size is certainly a major limitation of this study, as well as the heterogeneity in the severity of scoliosis and the time under brace treatment. Future studies should enroll a larger and more homogeneous sample. When interpreting our results, it should be noted that all the girls included in our sample underwent physical therapy and brace treatment. Thus, we cannot exclude that the increased attention towards shoulders and backs could be a consequence of treatment per se. Indeed, many physical therapy exercises aim to increase shoulder and back muscle awareness, leading to higher attention towards these body parts [38]. Future studies should assess attentional bias in girls with scoliosis before any treatment to rule out this possibility.

## 6. Conclusions

Our results provide preliminary evidence of body image dissatisfaction and attention bias towards disease-relevant body parts in girls with AIS. Assessing and monitoring the presence of cognitive biases in adolescent girls affected by idiopathic scoliosis has clinical relevance, as they can contribute to the maintenance and/or exacerbation of body image disorders. Targeting dysfunctional cognitive processes associated with body image disorders is fundamental to preventing the insurgence of comorbid psychopathologies (i.e., anxiety, depression, eating disorders, sleep, and substance abuse disorders) in at-risk populations, such as AIS. Thus, attentional bias examination and modification might be a promising avenue for intervention research. To achieve this, psychological assessment should be integrated with routine AIS evaluations and ad hoc cognitive behavioral interventions (e.g., attention bias modification treatments) and psychological support should be provided as needed. Girls with AIS could benefit from targeted psychotherapy to avoid developing any severe psychopathology and increase their self-confidence and life quality.

## Figures and Tables

**Table 1:** Sample descriptive characteristics.

Subject ID	Group	Age (Years)	Weight (kg)	Height (m)	BMI
1	AIS	17.1	53.5	1.69	18.73
2	AIS	13,7	52	1.62	19.94
3	AIS	13,9	44	1.61	17.08
4	AIS	14,1	62	1.75	20.24
5	AIS	15,7	66	1.64	24.69
6	AIS	14	57	1.65	20.94
7	AIS	15	45,5	1.71	15.65 *
8	AIS	16.3	38	1.48	17.34
9	AIS	13.6	54	1.63	20.32
10	AIS	14	54	1.48	24.65
11	AIS	15.1	54	1.64	20.2
12	AIS	15.7	44	1.62	17
13	AIS	14.2	54	1.64	20.08
14	AIS	15.9	44	1.58	17.63
1	Control	14.7	84,5	1.6	33.64 **
2	Control	14	48,5	1.6	19.8
3	Control	14	48,5	1.6	18.83
4	Control	11.6	50	1.5	20.95
5	Control	13.4	52	1.6	21.37
6	Control	15.5	52	1.6	20.44
7	Control	14.8	67	1.6	25.22
8	Control	15.5	45	1.6	17.47
9	Control	14.2	52	1.8	17
10	Control	14.6	56	1.6	21.14
11	Control	13.7	50	1.7	17.3
12	Control	13.6	49	1.6	20.26
13	Control	16.6	70	1.7	24.39
14	Control	14.9	50	1.6	20.16

* Excluded for BMI < 17; ** Excluded for BMI > 25.

**Table 2:** Anamnestic information of the idiopathic scoliosis group.

Subject ID	Brace (Months)	Cobb (deg)	Curve Site	Curve Lateralisation	Risser Sign (%)
1	48	20	Thoracic	right	100
2	20	21	Thoracic	right	80
3	30	38	Thoracic	right	100
4	1	43	Thoracic	right	75
5	19	21	Lumbar	right	100
6	4	24	Thoracic	right	40
7	19	25	Thoracolumbar	right	90
8	7	55	Thoracic	right	85
9	1	25	Lumbar	right	10
10	65	22	Thoracic	right	100
11	27	27	Lumbar	left	60
12 *	1	24	Lumbar	left	100
13	30	28	Lumbar	left	85
14	41	28	Lumbar	left	100

* Excluded for BMI < 17.

## Data Availability

The datasets generated and/or analyzed during the current study are not publicly available due to participants’ privacy. Anonymous data and code will be made available upon reasonable request to the corresponding author. None of the experiments was pre-registered.
